# Excess mortality in COVID-19-negative people with non-communicable disorders during the first pandemic wave

**DOI:** 10.1186/s12889-025-21782-9

**Published:** 2025-02-22

**Authors:** Daniel V. Vigo, Kristen L. Hudec, Saimoom Ferdous, Richard J. Munthali, Julia Pei, Lonna Munro, Krishna Pendakur, Lakshmi N. Yatham, Ronald C. Kessler, Raymond T. Ng, Joseph H. Puyat

**Affiliations:** 1 School of Population and Public Health, University of British Columbia, 2206 East Mall, Vancouver, BC V6T 1Z3 Canada; 2 Department of Psychiatry, Faculty of Medicine, University of British Columbia, 2255 Wesbrook Mall, Vancouver, BC V6T 2A1 Canada; 3 Department of Global Health and Social Medicine, Harvard Medical School, 641 Huntington Avenue, Boston, MA 02115 USA; 4 Department of Economics, Simon Fraser University, 8888 University Drive, Burnaby, BC V5A 1S6 Canada; 5 Department of Health Care Policy, Harvard Medical School, 180 Longwood Avenue, Boston, MA 02115 USA; 6 Department of Computer Science, University of British Columbia, 2366 Main Mall, Vancouver, BC V6T 1Z4 Canada; 7 Centre for Health Services and Policy Research, School of Population and Public Health, University of British Columbia, 2206 East Mall, Vancouver, BC V6T 1Z3 Canada

**Keywords:** COVID-19, SARS-CoV-2, Pandemic, Excess mortality, Morbidity, Cohort study

## Abstract

**Background:**

Estimating the indirect mortality due to COVID-19 is of the utmost importance to develop adequate public health policy during future outbreaks.

**Methods:**

From province-wide administrative datasets, we identified British Columbians who tested negative for COVID-19 during the first wave and never tested positive throughout 2020. We obtained a pre-pandemic (2018) cohort matched on age, sex, history of non-communicable disorders (NCDs), multimorbidity, and severity/acuity, and implemented a doubly robust estimation of the effect of the first pandemic wave on mortality.

**Results:**

The adjusted odds ratio (AOR) of death was 3.2 times higher for a 2020 cohort who tested negative for COVID-19 (*n* = 123,133), compared to matched pre-pandemic controls. In both cohorts, a majority (72.5%) experienced at least one pre-existing NCD. Stratification by NCD shows an AOR of death ranges between 2–for people with substance use disorders– and 7–for people previously undiagnosed with NCDs (e.g., incident cases that went untreated). The largest subgroup was composed of people with mental disorders (47,413 people), with an AOR of death of 2.5. Though the COVID-19 direct mortality in the general population remained low (1.9 per 10,000), the excess mortality in this COVID-negative cohort was extremely high − 4,085 of the 123,133– which entails a minimum indirect excess mortality death rate of 6.5 per 10,000 in the general population.

**Conclusions:**

During the first pandemic year, mortality in COVID-negative adults was several times greater than before COVID-19, in people with matched NCD distribution and severity. Our findings suggest that low direct COVID-19 mortality was accompanied by less visible–but much higher– indirect mortality due to undiagnosed and/or untreated NCDs, highlighting the need to focus not only on mitigating the harms of new agents, but also of continuing service delivery for treatable conditions.

**Supplementary Information:**

The online version contains supplementary material available at 10.1186/s12889-025-21782-9.

## Introduction

Several commentators have pointed out that estimating excess all-cause mortality is the most appropriate way to gauge the toll of the coronavirus disease (COVID-19) pandemic. While excess mortality denotes the additional deaths during the pandemic (related to expected deaths based on historical trends), all-cause mortality captures not only direct mortality but also indirect mortality from the potential impact of the pandemic context and restrictions (either self-imposed to prevent exposure to the virus or imposed by public health orders) on help-seeking behaviours for non-COVID-related disorders (e.g., delayed diagnosis, cancelled surgeries, unavailable emergency services) [1–3]. The estimated global excess mortality associated with COVID-19–either directly or indirectly– reached 14.9 million globally in 2020 and 2021 [4]. More specifically, a study puts excess deaths due to COVID-19 in our area, British Columbia (BC), Canada, at 9,496 from March 2020 to October 2021 [5]. It is, however, impossible to discriminate in these studies which fraction of excess all-cause mortality is due to untested COVID-positive individuals (i.e., unreported but directly related to COVID-19) and which is due to other disorders in COVID-negative people (i.e., indirectly related to the COVID-19 pandemic).

Our goal was to measure the impact of the first pandemic wave (and the accompanying restrictions) on mortality among people who (a) did not get infected with COVID-19, but (b) had other disorders that required medical care. In order to do this, we identified people in BC who tested negative for COVID-19 throughout this period (this directly ensures compliance with criterion (a), and indirectly with (b) due to the policies that initially restricted testing to people with significant pre-existing morbidity, advanced age, or severe clinical deterioration), and we compared their mortality to an equivalent pre-pandemic cohort. Our main interest was the period of maximum health service restrictions, hence our focus on the first pandemic wave, as this was the period with the most stringent lockdown in BC. Specifically, we wanted to understand the distribution of excess mortality indirectly associated with the COVID-19 pandemic and ensuing health system shutdown by examining confirmed COVID-negative individuals with and without pre-existing non-communicable disorders (NCDs).

## Methods

### Sample

We analyzed administrative data curated by Population Data BC capturing all health care utilization of BC adult residents in two retrospective cohorts, one for 2018 (pre-pandemic cohort) and one for 2020 (pandemic cohort) [6]. Population Data BC provides secure access to pre-approved, de-identified, linked data from federal and provincial agencies, including BC Ministry of Health and BC Vital Statistics Agency [7]. Information entered into individual health care records by service providers or agencies (e.g., hospital, coroner, pharmacy, insurance) is compiled and routinely updated (typically every 3 to 6 months) by Population Data BC to ensure accuracy and completeness. Similar to other types of data, administrative data has certain limitations, such as human error at the point of entry (e.g., incorrect selection in the medical chart) or delays in reporting (e.g., smaller organizations may take longer to verify and share data), but our data have four features worth highlighting in comparison with other health transaction data. First, our dataset forms a panel for the entire adult population in contact with health services, rather than a special subset of the population (e.g., Veteran’s Administration participants, hospital data). Consequently, there is no bias introduced by selection into the dataset from which the cohorts of interest were extracted (i.e., our sample contains the universe of people in BC). As such, we can characterize all-cause mortality during the first wave of the pandemic among uninfected adults in BC and possibly provide a useful comparator for similar adult populations from comparable pandemic contexts. Second, we have access to individual (de-identified) health records directly from multiple sources, which allows us to construct a comprehensive historical and current record for the individuals within our dataset. Additionally, in many instances, we have several sources with which to verify a datapoint; for example, if a patient dies in hospital, our data include record of their death in the hospital file (discharge status = deceased) as well as the vital death statistics file (date of death documented by the coroner) and the provincial registration/consolidation file (flag to denote death during the reporting period), so our dataset allows us to confirm incidents of interest (i.e., death, COVID infection) in the current study. Third, we can distinguish COVID-negative from COVID-positive people among those tested during the first pandemic wave. Fourth, because these are panel data that follow people over time, we can construct measures of pre-existing NCD morbidity and severity using past medical records for a given person and use these variables (amongst others) to construct a control sample that is actually comparable to our “treatment” sample.

All living, active residents (as determined through the provincial death registry file and by any interaction with the health system within the year of interest or previous 2 years; i.e., 2016–2018 for 2018), who were at least 18 years old on January 1st in each year, were initially included in the cohorts (2018: *n* = 4,147,274; 2020: *n* = 4,224,839). We accessed all episodes of hospital [8], emergency [9], and outpatient physician services in the province along with all COVID-19 laboratory test results in 2020 plus data on deaths in the province [10–12]. 

We first identified all people in BC who tested negative on a polymerase chain reaction (PCR) test for COVID-19 during the first pandemic wave (which ended on June 8, 2020), then we removed anyone who tested positive for COVID-19 at any subsequent point in 2020, creating a cohort consisting of all people who tested negative one or more times during the COVID-19 first wave (*n* = 123,133) and never tested positive for the rest of the year (to exclude any deaths related to subsequent infection). Of note, given the stringent testing policies in BC during the pandemic onset, people who were tested early can be expected to have higher morbidity and severity, regardless of their COVID-19 status: until well into April 2020, people with non-severe COVID-19 symptoms were told to isolate at home, and testing was restricted to those likely to be hospitalized, healthcare workers, and residents of long-term care facilities [13, 14]. These testing policies gradually evolved to include people with conditions predisposing them to severe symptoms (such as old age and comorbidities), and finally to anyone with the usual list of COVID-19 symptoms.

We then created a non-overlapping “pre-pandemic cohort” from the 2018 universal sample using propensity score matching. To begin, we created propensity scores for each individual in the 2020 cohort and 2018 universal sample. Variables for propensity scores were based on demographic (i.e., age, sex) and clinical characteristics (i.e., pre-existing NCDs; see Supplementary Table 1, Additional File 1 for a list of disorders and diagnostic codes included by condition) that have been reported to contribute to differences in mortality rate and on available health administration data pertaining to these characteristics. We also created a dummy variable for multi-morbidity (more than two of the pre-existing NCDs included), which is known to be common among patients with the focus conditions and to be associated with further increased mortality risk [15]. Given the stringent testing policies mentioned above, matching only on age, sex, NCD, and multimorbidity could fail to reproduce the severity of the 2020 cohort, so we also included severity and acuity variables (hospitalization days and intensive care unit, ICU, admission during the previous year) in our propensity score. Given our use of population-based data, no *p*-value or criterion-based variable selection procedures were employed. Next, we performed 1:1 propensity score matching, in which one case from the 2020 cohort was matched to one case from the 2018 sample. Matching was performed using the nearest neighbor method without replacement and a maximum caliper distance of 0.2 of the propensity score, meaning the case from 2018 with the propensity score closest to the case from 2020 (and not more than 0.2 difference) was matched and removed from the pool (i.e., not replaced) to create a non-overlapping matched cohort. Quality of the matching was evaluated by comparing the proportion of people with various demographic and clinical characteristics in both the 2020 pandemic and 2018 pre-pandemic groups (Table 1).

Since COVID-19 cases in BC emerged during early 2020 and peaked during March and April, with a post-first wave valley during June and July, we considered deaths occurring during or within six months of the first wave as potentially related to decreased availability of routine services, so we included all deaths throughout both target years.

### Statistical analysis

We compared the composition of both cohorts (e.g., demographic characteristics, pre-existing NCD morbidity) to ensure well-matched groups, then computed the mortality counts and rates (number of deaths within the group divided by the total number of people within the group) for each cohort and subgroup. An *excess* mortality rate was determined by subtracting the pre-pandemic (2018) cohort mortality from the pandemic (2020) cohort mortality, providing a measure of all-cause mortality in 2020 relative to 2018 (i.e., pandemic deaths in excess of anticipated based on pre-pandemic deaths). For ease of comparison, mortality rates are expressed as deaths per 10,000. In order to examine the odds of death, particularly by cohort or subgroup, we used regression models to determine the adjusted odds ratios (AOR; the probability of death divided by the probability of surviving). We first estimated a multivariable logistic regression model with a dummy variable for “pandemic year” (i.e., 2020 vs. 2018) as the independent variable and death as the dependent variable. We implemented a doubly robust estimation by also adjusting for the covariates used in the propensity score model (i.e., age, sex, each NCD, multimorbidity, hospitalization days, and admission to ICU) [16]. We then estimated stratified models, including in a stratum only people with each NCD; with multimorbidity; and, without any previously diagnosed NCD, also with a doubly robust approach controlling for age, sex, all comorbidities, hospitalization days, and ICU admission. To perform data analysis and propensity score matching, we used the Python programming language version 3.5.3 (Python Software Foundation) and the following libraries: SciPy [17], Pandas [18], NumPy [19], Matplotlib [20], Seaborn [21], scikit-learn [22], and pscore_match [23]. For the statistical tests, we used the Python package Statsmodels and SPSS statistical software version 26.0 (IBM Corporation).

## Results

Comparison of pre-pandemic and pandemic cohort composition as well as their mortality for each stratum (Table 1) indicates that the death rate for these propensity score-matched cohorts was 210.1 per 10,000 in 2018 and 541.9 per 10,000 in 2020. This indicates an excess mortality rate of 331.8 per 10,000 for a cohort of people who were free of COVID-19 throughout the whole period, as confirmed by one or more PCR tests. The largest subgroup was people with a mental disorder (*n* = 47,490 and 47,413 respectively for 2018 and 2020), whose deaths increased from 367.0 to 749.4 per 10,000, an excess death rate of 382.4 per 10,000 in this stratum. The largest number of deaths occurred in people with cardiovascular disorders, which increased from 451.0 to 1,088 deaths per 10,000, an excess death rate of 637.2 per 10,000 in this stratum.

**Table 1:** Pandemic COVID-negative and pre-pandemic matched cohort composition and mortality (*n* = 123,133 for both cohorts)

	Pre-pandemic Cohort (2018)	Pandemic Cohort (2020)	Pre-pandemic Cohort (2018)	Pandemic Cohort (2020)
	Subgroup Characteristics *n* (% of cohort)		Subgroup Deaths *n* (% of cohort)	
**Sex**				
Female	74,021 (60.1)	74,011 (60.1)	1,242 (1.0)	3,030 (2.5)
Male	49,112 (39.9)	49,122 (39.9)	1,345 (1.1)	3,642 (3.0)
**Age in Years**				
18–40	43,023 (34.9)	43,043 (35.0)	80 (0.1)	193 (0.2)
41–60	41,774 (33.9)	41,773 (33.9)	239 (0.2)	704 (0.6)
61–70	16,248 (13.2)	16,288 (13.2)	327 (0.3)	943 (0.8)
71–80	10,968 (8.9)	10,972 (8.9)	513 (0.4)	1,555 (1.3)
81+	11,120 (9.0)	11,057 (9.0)	1,428 (1.2)	3,277 (2.7)
**Disorder Category** ^a^				
No Previous NCD Morbidity	33,889 (27.5)	33,889 (27.5)	51 (< 0.1)	322 (0.3)
Immune System-Related	5,045 (4.1)	5,092 (4.1)	239 (0.2)	577 (0.5)
Cardiovascular	46,478 (37.7)	46,426 (37.7)	2,096 (1.7)	5,052 (4.1)
Endocrine & Metabolic	37,098 (30.1)	37,093 (30.1)	1,604 (1.3)	3,650 (3.0)
Cancer	17,159 (13.9)	17,179 (14.0)	998 (0.8)	2,230 (1.8)
3 or More Morbidities	29,029 (23.6)	29,019 (23.6)	1,968 (1.6)	4,155 (3.4)
Mental Disorders	47,490 (38.6)	47,413 (38.5)	1,743 (1.4)	3,553 (2.9)
Chronic Respiratory Diseases	21,305 (17.3)	21,301 (17.3)	1,210 (1.0)	2,446 (2.0)
Chronic Kidney Diseases	7,647 (6.2)	7,672 (6.2)	926 (0.8)	1,701 (1.4)
Chronic Liver Diseases	3,827 (3.1)	3,851 (3.1)	238 (0.2)	428 (0.3)
Substance Use Disorders	8,199 (6.7)	8,210 (6.7)	414 (0.3)	733 (0.6)
**Severity Category** ^b^				
No Hospitalization in Prior Year	107,528 (87.3)	107,497 (87.3)	1,003 (0.8)	3,885 (3.2)
Hospitalized in Prior Year	15,605 (12.7)	15,636 (12.7)	1,584 (1.3)	2,787 (2.3)
1–27 Days Hospitalized in Prior Year	12,592 (10.2)	12,579 (10.2)	908 (0.7)	1,952 (1.6)
≥28 Days Hospitalized in Prior Year	3,013 (2.4)	3,057 (2.5)	676 (0.5)	835 (0.7)
No ICU in Prior Year	121,166 (98.4)	121,142 (98.4)	2,350 (1.9)	6,248 (5.1)
With ICU in Prior Year	1,967 (1.6)	1,991 (1.6)	237 (0.2)	424 (0.3)

^a^Individuals with multiple pre-existing chronic disorders are included in all applicable disorder categories, so percentages sum to greater than 100

^b^Based on hospital admissions in the year prior to the exposure or reference year (i.e., 2017 for the pre-pandemic cohort, 2019 for the pandemic cohort). Individuals are included only once per category (i.e., hospitalization or no hospitalization, ICU or no ICU), so percentages sum to 100

ICU = intensive care units. NCD = non-communicable disorder

The first multivariable logistic regression model includes the full pre-pandemic and pandemic cohorts and gives an estimated adjusted odds ratio of 3.2 on the variable “pandemic year” (Table 2). This means the adjusted odds of death (AOR, the probability of death divided by the probability of surviving) was 3.2 times higher in people who tested negative for COVID-19 in 2020 than in a matched 2018 cohort, controlling for sex, age, and chronic disease morbidity and severity. Stratified analyses showed that people with each NCD had increased odds of dying during or following the pandemic onset of between 2.0 (with substance use disorders) and 3.1 (with disorders affecting the immune system) times their pre-pandemic odds, in models controlling for all other NCDs.

**Table 2:** Multivariable logistic regressions describing the overall and chronic disorder-specific odds of dying during the pandemic year (2020) relative to a pre-pandemic year (2018)

		95% Confidence Interval	
**Multivariable model** ^a^	**AOR**: **Predictor in row**	**Lower**	**Upper**
Pandemic year (2020) vs. Pre-pandemic year (2018)	3.22	3.06	3.38
Female vs. Male	0.66	0.63	0.69
Age 41–60 years vs. < 41 years	3.05	2.66	3.50
Age 61–70 years vs. < 41 years	8.61	7.51	9.88
Age 71–80 years vs. < 41 years	19.30	16.86	22.08
Age 81 + years vs. < 41 years	51.39	45.02	58.67
**Multivariable models stratified by specific** **pre-existing chronic disorder** ^b^	**AOR**: **Pandemic year**	**Lower**	**Upper**
No Previous NCD Morbidity	6.96	5.15	9.41
Immune System-Related	3.11	2.62	3.68
Cardiovascular	3.04	2.87	3.22
Endocrine & Metabolic	2.88	2.69	3.07
Cancer	2.84	2.61	3.09
3 or More Morbidities	2.60	2.45	3.76
Mental Disorders	2.50	2.34	2.67
Chronic Respiratory Diseases	2.48	2.30	2.68
Chronic Kidney Diseases	2.28	2.08	2.50
Chronic Liver Diseases	2.13	1.78	2.55
Substance Use Disorders	1.99	1.75	2.27

^a^Adjusted for sex, age, various pre-existing chronic disorders, hospitalization, and intensive care unit admission

^b^Adjusted for sex, age, hospitalization, intensive care unit admission, and pre-existing chronic comorbid disorders other than the condition used for stratification (only the AOR for pandemic year as a predictor is presented); the stratified model for no previous NCD morbidity was adjusted for sex and age only

Note. In these regressions, the coefficient on “pandemic year” gives the difference in log-odds for death in the 2020 versus 2018 cohort, holding constant the control variables. If these coefficients exceed one, then the people who tested negative for COVID-19 died with a higher probability in 2020 than similar people in 2018. AOR = adjusted odds ratio. NCD = non-communicable disorder

Surprisingly, the largest increase in death rate–a six-fold increase– and the largest AOR (7.0) occurred among people free of any previously diagnosed NCD. Indeed, their mortality rate jumped from 15.0 to 95.0 per 10,000 between 2018 and 2020. This group is of particular interest, because although all deaths analyzed here represent excess deaths among COVID-negative people, those without any previously detected NCD represent, in principle, people who were healthy until the previous year. Indeed, the mortality rate in this stratum is the lowest in both cohorts. Table 3 shows the causes of death for these relatively healthy individuals, as well as for people with a history of NCDs. As could be expected given the lockdown, deaths due to accidents plummeted [24], potentially explaining the decreased death rate in youth.

**Table 3:** Manner and age of death, by presence of chronic non-communicable disorders

	Pre-pandemic Cohort (2018)	Pandemic Cohort (2020)
**People without non-communicable disorders in previous 2 years**		
**Total Deceased**	51 / 33,889 (0.2%)	322 / 33,889 (1.0%)
**Manner of Death**		
Accident	≤ 5 / 51 (NA)	≤ 5 / 322 (NA)
Natural	42 / 51 (82.4%)	282 / 322 (87.6%)
Suicide	≤ 5 / 51 (NA)	≤ 5 / 322 (NA)
Homicide	0 / 51 (0.0%)	0 / 322 (0.0%)
Pending/Unknown	≤ 5 / 51 (NA)	33 / 322 (10.2%)
**Age in Years**		
18–40	10 / 51 (19.6%)	30 / 322 (9.3%)
41–60	9 / 51 (17.6%)	73 / 322 (22.7%)
61–70	7 / 51 (13.7%)	85 / 322 (26.4%)
71–80	8 / 51 (15.7%)	71 / 322 (22.0%)
81+	17 / 51 (33.3%)	63 / 322 (19.6%)
**Severity Category**		
No Hospitalization in Prior Year	50 / 51 (98.0%)	319 / 322 (99.1%)
1–27 Days Hospitalized in Prior Year	≤ 5 / 51 (NA)	≤ 5 / 322 (NA)
≥28 Days Hospitalized in Prior Year	≤ 5 / 51 (NA)	≤ 5 / 322 (NA)
Hospitalization in ICU in Prior Year	0 / 51 (0.0%)	0 / 322 (0.0%)
**People with any non-communicable disorders in previous 2 years**		
**Total Deceased**	2,536 / 89,244 (2.8%)	6,350 / 89,244 (7.1%)
**Manner of Death**		
Accident	95 / 2,536 (3.7%)	55 / 6,350 (0.9%)
Natural	2,378 / 2,536 (93.8%)	5981 / 6,350 (94.1%)
Suicide	15 / 2,536 (0.6%)	≤ 10 / 6,350 (NA)
Homicide	0 / 2,536 (0.0%)	≤ 5 / 6,350 (NA)
Pending/Unknown	48 / 2,536 (1.9%)	303 / 6,350 (4.8%)
**Age in Years**		
18–40	70 / 2,536 (2.8%)	163 / 6,350 (2.6%)
41–60	230 / 2,536 (9.1%)	631 / 6,350 (9.9%)
61–70	320 / 2,536 (12.6%)	858 / 6,350 (13.5%)
71–80	505 / 2,536 (19.9%)	1,484 / 6,350 (23.4%)
81+	1,411 / 2,536 (55.6%)	3,214 / 6,350 (50.6%)
**Severity Category**		
No Hospitalization in Prior Year	953 / 2,536 (37.6%)	3,566 / 6,350 (56.2%)
1–27 Days Hospitalized in Prior Year	907 / 2,536 (35.8%)	1,949 / 6,350 (30.7%)
≥28 Days Hospitalized in Prior Year	676 / 2,536 (26.7%)	835 / 6,350 (13.1%)
Hospitalization in ICU in Prior Year	237 / 2,536 (9.3%)	424 / 6,350 (6.7%)

Note. Small cell counts (≤ 5) are suppressed per Population Data BC guidelines to reduce risk of individual re-identification. Suicides were reported as ≤ 10 to preclude deduction of the number of homicides. As a result, group categories may not sum to 100%. ICU = intensive care units. NA = not available

**Fig. 1:**
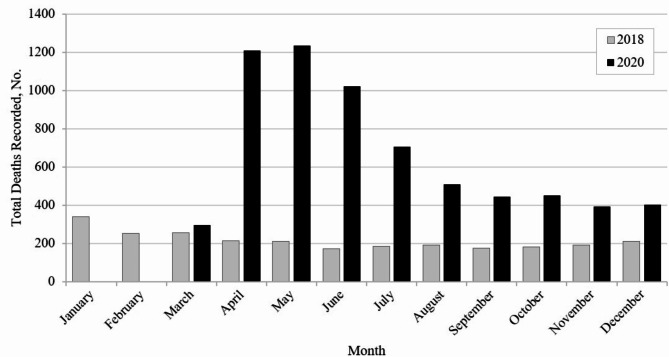
Total deaths among individuals tested negative for COVID-19 in 2020 and matched cohort in 2018

Finally, Fig. 1 shows that total deaths in 2020 escalated rapidly, reached a high plateau between March and July, and remained higher throughout the observation period.

Reflects total deaths in British Columbia, Canada during the cohort year (2020 or 2018). In 2020, by cohort definition, everyone survived at least until they were tested, so there are no deaths in January and < 5 deaths in February.

## Discussion

Our results indicate that during the first pandemic wave and the ensuing months, the death rate of people who were free of COVID-19 in BC (i.e., always tested negative) more than tripled that of a matched pre-pandemic cohort. In absolute terms, the group with the largest increase consisted of those with cardiovascular disorders, but in relative terms, the largest increase occurred among people who were not only COVID-negative but also had no previously diagnosed NCD, signaling that some of them may represent incident cases that escalated to death before routine detection and treatment was provided. The spike in deaths among COVID-negative people seemed to coincide with the period of almost total health system shutdown for non-COVID-related complaints.

Our results point to a disturbing aspect of the context surrounding the pandemic onset. During early 2020, against a backdrop of decimation as COVID-19 swept through northern Italy, most well-functioning health systems made the rational decision to focus all resources on mitigating the harms of COVID-19. In BC, this goal was largely achieved during 2020: there were 957 deaths due to COVID-19, a death rate of 1.9 per 10,000 in the general population (957/5,155,495*10,000). However, largely unseen were the 4,085 excess deaths in people that tested negative for COVID-19 during the same period, as compared to a matched 2018 cohort. This excess mortality in our cohort of COVID-negative individuals (4,085 of 123,133 people) suggests indirect mortality in the general population in 2020 was, at a minimum, 6.5 per 10,000 (three times larger than the COVID-19 specific mortality rate). We are unable to estimate how many people would have died of COVID-19 if routine health services for NCDs had been maintained. In all likelihood, potentially thousands of COVID-19 deaths were averted by the restrictions on routine health services. We are also unable to estimate how many of the indirect excess deaths we have identified would have been avoided if less restrictive measures had been put in place, as overwhelmed health systems cannot properly treat even well-known disorders [25]. 

Our results highlight the dual responsibility that health systems have in the face of COVID-19 and other major epidemics or pandemics: in addition to minimizing the excess deaths due to the new agent, of equal–potentially greater– importance is minimizing the excess deaths due to existing, well understood, treatable disorders. Many countries of all income levels seem to have fallen short at both tasks. COVID-19 deaths long ago surpassed 1,000,000 in the United States [26], and the excess deaths resulting from untreated NCDs remain largely unknown [27]. 

Whereas many jurisdictions succeeded in keeping COVID-specific excess mortality low, the performance of well-organized health systems with respect to adequately balancing the two aforementioned goals is less clear. With this in mind, the stratum of people without any of the NCDs we investigated– but the highest relative odds of dying– are of interest. Our interpretation is that these people may have developed an NCD during the pandemic onset, which went untreated and led to such a large increase in mortality. The manner of death among this group was unremarkable, meaning there were not unusually high numbers of a specific cause of death (e.g., suicide, homicide) as might be expected. Although accident-related deaths were substantially lower in 2020, likely due to pandemic restrictions, most deaths were still due to natural causes, as would occur with an undiagnosed or untreated disorder. In addition to undetected NCDs, deaths may have resulted from medical emergencies that occurred suddenly without prior indication of pre-existing conditions, such as ruptured appendix, myocardial infarction, anaphylaxis, or choking; and, if symptoms were not readily apparent to others (e.g., headache, confusion) or if help was limited (i.e., person alone and incapacitated) or delayed (e.g., lengthy ambulance response times, medication shortages), these events could result in death for seemingly healthy individuals. An alternative interpretation is that these individuals may have had pre-existing disorders that we did not investigate. However, the fact that the mortality rates for this group in both the 2018 and 2020 cohorts (0.2% and 1.0%, respectively) are the lowest, by far, of all disorder strata we investigated argues in favor of our interpretation. Other factors, including environmental (e.g., food insecurity, unhoused or unsheltered, lacking adequate heating/cooling in peak weather periods, lack of telehealth options) and sociocultural (e.g., not disclosing health issues to avoid being a burden to others, not seeking in-person medical help or testing due to fear of COVID-19 infection), also warrant investigation, though some may be challenging to ascertain. While the majority in both cohorts (73%) had at least one NCD, this group of COVID-negative people without previously diagnosed NCDs may be those most uniquely and inadvertently affected by the disruption to the health system; health systems must account for this group, and others at particularly high risk, in planning for future outbreaks to both avoid overtaxing health systems and prevent excess deaths of this magnitude in treatable conditions.

Excess mortality in the pandemic cohort was evident, but deaths varied across sociodemographic factors, with mortality lower in females and progressively higher as age increased. These findings are not entirely surprising, and may be due to sex and age differences in the presentation and identification of health conditions generally, wherein males and older individuals may be at risk for emerging disorders and/or circumstances that may lead to death, particularly amidst a pandemic. For example, greater reluctance to seek help or greater chance of experiencing a high-risk event (e.g., myocardial infarction) may result in serious health decline without intervention. Moreover, middle-aged and older adults may be especially likely to develop disorders that increase risk of death, and while everyone was impacted by the start of the pandemic, the physical and emotional toll of social isolation may have been heightened for these age groups and potentially detrimental to their health. Another consideration is the composition of our sample; females are over-represented in our cohorts (60%) but also in the healthcare professions. Given the requirement for regular testing for healthcare (e.g., hospital, long-term care facilities) employees during this period, a disproportionate number of females are likely healthcare workers, who may be somewhat insulated from the effects of a health system shutdown or reduction in health services, since they remained in contact with the health system via their role as a healthcare worker; as such, they may have been able to seek help for ailments as they developed (rather than delaying or foregoing care) and thus had lower mortality. With health systems experiencing high turnover and staffing shortages, future investigations should explore sociodemographic differences across professions to better understand and mitigate potential mortality risk.

It should be noted that our analysis does not include excess deaths in people who were not tested for COVID-19, because it would be impossible to ascertain who died free of COVID-19 and who died following an untested infection. As we pointed out earlier, the increased likelihood of dying ascertained in people who tested negative for COVID-19 cannot be extrapolated to the untested population, because the untested BC population can be expected to be healthier than our 2020 COVID-19 test-negative cohort, and to require less of the routine health services disrupted by the pandemic. However, given that the untested population is the largest subgroup (about 40 times those that tested negative) and the least likely to be infected (they remained untested even after expansion of testing policies), they are also likely to have contributed additional excess deaths in people without COVID-19, which would make our overall excess mortality estimate a lower bound.

Given our hypothesis that lack of routine health services during the first wave of the pandemic resulted in untreated disorders, leading to clinical deterioration and death, we regarded hospitalization and ICU admission during the observation period (i.e., current severity and acuity) as variables in the causal pathway and not as confounders that should be accounted for in the creation of the analytic cohorts. However, there were patients from the 2020 cohort that were already very sick (i.e., admitted to hospital) at the time of testing and this might have an undue influence on the estimated average effect. To examine how severity or acuity at the time of COVID-19 testing might have affected our findings, we conducted a sensitivity analysis where we removed people who were tested for COVID-19 while hospitalized (*n* = 14,767). This sensitivity analysis produced an overall AOR of 2.3 (Supplementary Table 2, Additional File 1), which is somewhat lower but still represents a large effect. This approach is likely overly stringent considering we are excluding those who were most impacted by the restriction of health services. Nevertheless, results from this sensitivity analysis on the remaining 108,366 who had no such “current” severity/acuity confirms the presence of a large effect.

Our study has several limitations. First, propensity score matching allows for the creation of a matched control group based only on observables, and unobserved variables may explain difference in outcomes. To mitigate this potential bias, we took several steps. Primarily, in addition to demographic characteristics and NCD morbidity, we included the severity and acuity measures in the construction of the control group. Supplementary Table 3, Additional File 1 shows the results of the model without including the severity and acuity variables: the AOR decreases from 3.5 without these measures to 3.2 in our main analysis, indicating that some bias was present due to unmeasured confounders (i.e., correlated missing regressors). However, the small magnitude of the change (about 10%) combined with the detail and strength of our severity and acuity measures suggest that our estimates may approximate the true effect. For example, suppose that the only correlated missing regressor in our initial regression was “true severity/acuity”. Our proxy for “true severity/acuity” is based on previous year hospitalization and ICU admission. We can decompose “true severity/acuity” into our proxy and an unmeasured component. Assuming that our proxy is reasonably good, it has the same scale and covariance with included regressors as the unmeasured component. Then, including our proxy in the initial model would reduce the bias caused by confounding severity/acuity by half [28]. Other confounders may exist that have not been included in our current analysis, because the information was not captured in the available administrative health data (e.g., lifestyle factors, socioeconomic status). Future studies may investigate the potential influence of these elements, though the impact of lifestyle factors may be captured, to some extent, by our measure of chronic and comorbid disorders, given the link between lifestyle characteristics and health conditions.

A second limitation is that our analysis was anchored in 2018, which means that any unusual circumstances affecting mortality during that year would affect our conclusions. To contextualize our findings, Supplementary Table 4, Additional File 1 shows the overall mortality in BC during the past decade. The number of deaths was increasing, plateaued between 2017 and 2019, spiked in 2020 and kept increasing in 2021. Also, our approach of including deaths from January through December in both target years makes our estimate of the excess mortality increase in 2020 conservative, as by our definition, no deaths occurred until people started getting tested for COVID-19 in February 2020.

Finally, we examined fine-grained descriptive data on time of death and cause of death. Excess deaths among people that tested negative for COVID-19 were, in general, due to “natural causes” and heavily concentrated in the months of March to July 2020, the peak months of health system shutdown. Although the 6-fold increase in deaths among people in the group without known disorders might be thought to be driven by a relatively small number of deaths, the magnitude of the odds-ratio for this group, which was higher than among individuals with any chronic conditions, and the non-overlapping confidence intervals argue against this interpretation.

Our study has important implications for health systems planning and public health policy: it should help decision-makers gauge the tradeoffs of restricting health service access in the face of COVID-19 variants of lesser or unclear lethality, or of new pandemics. The double responsibility of health systems we have highlighted–mitigating excess mortality due to new noxious agents while also avoiding fully preventable excess mortality due to known treatable disorders– should always guide policy. Given our findings, if due to future COVID-19 waves or new pandemics, decision-makers need to consider a restriction of routine health services, a nuanced approach should be implemented to mitigate the foreseeable and preventable deaths highlighted in this study.

## Electronic supplementary material

Below is the link to the electronic supplementary material.

Supplementary Material 1

## Data Availability

The data that support the findings of this study are managed by Population Data BC and not publicly available. Access to data provided by the Population Data BC Data Steward(s) is subject to approval but can be requested for research projects through the Data Steward(s) or their designated service providers. The following data sets were used in this study: Consolidation File (MSP Registration & Premium Billing); COVID-19 Testing; Hospital Separations; Medical Services Plan (MSP) Payment Information File; National Ambulatory Care Reporting System; Vital Events Deaths. You can find further information regarding these data sets by visiting the Population Data BC webpage at: https://my.popdata.bc.ca/project_listings/20-179/collection_approval_dates. Inquiries regarding data access may be addressed to Population Data BC (info@popdata.bc.ca). All inferences, opinions, and conclusions drawn in this publication are those of the authors, and do not reflect the opinions or policies of the Data Steward(s).
