# Supplementary material for: Excess mortality in COVID-19-negative people with non-communicable disorders during the first pandemic wave
Source: BMC Public Health. 2025 Feb 22;25:736. doi: 10.1186/s12889-025-21782-9 (PMC11847353; doi:10.1186/s12889-025-21782-9)
Supplement: Supplementary file 1 — Supplementary Material 1 [file 12889_2025_21782_MOESM1_ESM.docx]

Supplementary Material

**Table of Contents**

[**Table 1.** Diagnostic Codes for Non-communicable Disorders Used as Morbidities and Covariates. 2](#_Toc103669133)

[**Table 2.** Multivariable Logistic Regressions Describing Odds of Dying, Excluding Matched Pairs Containing 2020 Individuals First Tested (Negative) for COVID-19 in Hospital 4](#_Toc103669134)

[**Table 3.** Multivariable Logistic Regressions Describing Odds of Dying, Without Previous Year Hospitalization and Intensive Care Unit Admission in the Propensity Score Matching or as Control Variables 5](#_Toc103669135)

[**Table 4.** Annual Total Deaths and Population in British Columbia, Canada in the Past Decade. 6](#_Toc103669136)

[**References** 7](#_Toc103669137)

# Table 1. Diagnostic codes for non-communicable disorders used as morbidities and covariates.

| **Condition Category with Inclusions and Associated Codes by Coding System**^a^ |
| --- |
| Cardiovascular Disorders |
| *Includes heart diseases (ischaemic, pulmonary, other forms), cerebrovascular diseases, hypertensive diseases, rheumatic fever and heart diseases* |
| ICD-9^1^: 390-398, 401-405, 410-417, 420-438, 440-444, 447-448, 451-459 |
| ICD-10^2^: I00-I02, I05-I09, I10-I15, I20-I28, I30-I52, I60-I69 |
| Endocrine, Metabolic, & Nutritional Disorders |
| *Includes disorders of thyroid gland, diabetes mellitus, disorders of glucose regulation, disorders of other endocrine glands, malnutrition and other nutritional deficiencies, obesity and other hyperalimentation, metabolic disorders (lactose intolerance, cystic fibrosis, amyloidosis, etc.)* |
| ICD-9: 240-246, 250, 251-273, 275-277, 278 |
| ICD-10: E00-E07, E10-E14, E15-E16, E20-E35, E40-E46, E50-E64, E65-E68, E70-E90 |
| Cancers |
| *Includes malignant neoplasms of any site, melanoma, in situ neoplasms, neoplasms of uncertain or unknown behavior* |
| ICD-9: 140-165, 170-175, 179-208, 230-239 |
| ICD-10: C00-C97, D00-D09, D37-D48 |
| Chronic Respiratory Disorders |
| *Includes chronic lower respiratory diseases (bronchitis, emphysema, other chronic obstructive pulmonary disease, asthma, etc.), lung diseases due to external agents, other respiratory diseases principally affecting the interstitium, suppurative and necrotic conditions of lower respiratory tract, other diseases of pleura* |
| ICD-9: 490-496, 500-508, 510-519 |
| ICD-10: J40-J47, J60-J70, J80-J86, J90-J94 |
| Chronic Kidney Disorders |
| *Includes chronic kidney disease, unspecified kidney failure* |
| ICD-9: 581-589 |
| ICD-10: N18-N19 |
| Chronic Liver Disorders |
| *Includes diseases of liver (chronic hepatitis, fibrosis and cirrhosis of live, other inflammatory liver diseases, etc.)* |
| ICD-9: 570-573 |
| ICD-10: K70-K77 |
| Immune System Related Disorders |
| *Includes inflammatory polyarthropathies (rheumatoid arthritis, psoriatic and enteropathic arthropathies, gout, etc.), systemic connective tissue disorders (polyarteritis, etc.), disorders involving immune mechanism (sarcoidosis, etc.), immunosuppression due to transplanted organ or tissue* |
| ICD-9: 274, 279, 446, V42 |
| ICD-10: M05-M14, M30-M36, D80-D89, Z94 |
| Mental Disorders |
| *Includes psychotic disorders (Schizophrenia, Schizotypal disorder, etc.), affective/mood disorders (manic episode, bipolar affective disorder, recurrent depressive disorder, etc.), anxiety and stress-related disorders (phobic anxiety disorders, obsessive-compulsive disorder, post-traumatic stress disorder, dissociative disorders, somatoform disorders, etc.), organic neurocognitive mental disorders (dementia, delirium, Alzheimer's disease, etc.), senility* |
| ICD-9: 290, 293-298, 300, 306, 307.8, 308-311, 331, 797, 50B^b^ |
| ICD-10: F00, F03-F07, F09, F20-F25, F28-F34, F38-F45, F48, G30-G32 (exclude G31.2), R54 |
| Substance Use Disorders |
| *Includes mental disorders due to use of psychoactive substance (alcohol, opioids, cannabinoids, sedatives or hypnotics, cocaine, other stimulants, hallucinogens, volatile solvents, multiple drug use), poisoning by narcotics and hallucinogens, toxic effects of alcohol, degeneration of nervous system due to alcohol* |
| ICD-9: 291, 292, 303-305 (including 3050, 3052-3059), 980 |
| ICD-10: F10-F16, F18, F19, T40, T42, T51, G31.2 |

^a^Health system data uses different coding systems within British Columbia. Diagnoses were screened using the appropriate code system for the particular dataset. People were considered to have a specific disorder if they had any encounter with the health system (as inpatient, outpatient or with emergency services) referencing that specific disorder category as a resulting diagnosis during the previous 2 years.

^b^Per the BC Ministry of Health,^3^ this is a BC-specific diagnostic code used by physician billing/insurance (Medical Services Plan) for both anxiety and depression.

#

# Table 2. Multivariable logistic regressions multivariable logistic regressions describing odds of dying, excluding matched pairs containing 2020 individuals first tested (negative) for COVID-19 in hospital (*n*=108,366 for both cohorts).

^a^Adjusted for sex, age, various pre-existing chronic disorders, hospitalization, and intensive care unit admission.

^b^Adjusted for sex, age, hospitalization, intensive care unit admission, and pre-existing chronic comorbid disorders other than the condition used for stratification; the stratified model for no previous NCD morbidity was adjusted for sex and age only.

NCD=non-communicable disorder.

|  | **AOR:**  **Predictor in row** | **95% Confidence Interval** | |
| --- | --- | --- | --- |
| **Multivariable model**^a^ |  | **Lower** | **Upper** |
| Pandemic year (2020) vs Pre-pandemic year (2018) | 2.33 | 2.18 | 2.49 |
| Female vs Male | 0.66 | 0.62 | 0.70 |
| Age 41-60 years vs <41 years | 2.90 | 2.43 | 3.47 |
| Age 61-70 years vs <41 years | 8.56 | 7.16 | 10.24 |
| Age 71-80 years vs <41 years | 22.55 | 18.92 | 26.88 |
| Age 81+ years vs <41 years | 69.17 | 58.28 | 82.09 |
| **Multivariable models stratified by specific**  **pre-existing chronic disorder**^b^ | **AOR:**  **Pandemic year** | **Lower** | **Upper** |
| No Previous NCD Morbidity | 3.60 | 2.57 | 5.04 |
| Immune System-Related | 2.14 | 1.69 | 2.72 |
| Cardiovascular | 2.23 | 2.07 | 2.41 |
| Endocrine & Metabolic | 2.10 | 1.92 | 2.30 |
| Cancer | 2.03 | 1.81 | 2.28 |
| 3 or More Morbidities | 1.96 | 1.81 | 2.12 |
| Mental Disorders | 2.10 | 1.93 | 2.27 |
| Chronic Respiratory Diseases | 1.74 | 1.57 | 1.94 |
| Chronic Kidney Diseases | 1.71 | 1.51 | 1.95 |
| Chronic Liver Diseases | 1.67 | 1.29 | 2.15 |
| Substance Use Disorders | 1.51 | 1.27 | 1.80 |

# Table 3. Multivariable logistic regressions describing odds of dying, without previous year hospitalization and intensive care unit admission in the propensity score matching or as control variables (*n*=123,133 for both cohorts).

^a^Adjusted for sex, age, and various pre-existing chronic disorders.

^b^Adjusted for sex, age, and pre-existing chronic comorbid disorders other than the condition used for stratification; the stratified model for no previous NCD morbidity was adjusted for sex and age only.

Note. In these regressions, the coefficient on “pandemic year” gives the difference in log-odds for death in the 2020 versus 2018 cohort, holding constant the control variables. If these coefficients exceed one, then the people who tested negative for COVID-19 died with a higher probability in 2020 than similar people in 2018. AOR=adjusted odds ratio. NCD=non-communicable disorder.

|  | **AOR:**  **Predictor in row** | **95% Confidence Interval** | |
| --- | --- | --- | --- |
| **Multivariable model**^a^ |  | **Lower** | **Upper** |
| Pandemic year (2020) vs Pre-pandemic year (2018) | 3.51 | 3.34 | 3.70 |
| Female vs Male | 0.66 | 0.63 | 0.69 |
| Age 41-60 years vs <41 years | 3.18 | 2.76 | 3.66 |
| Age 61-70 years vs <41 years | 9.03 | 7.84 | 10.39 |
| Age 71-80 years vs <41 years | 20.56 | 17.89 | 23.64 |
| Age 81+ years vs <41 years | 58.74 | 51.24 | 67.33 |
| **Multivariable models stratified by specific**  **pre-existing chronic disorder**^b^ | **AOR:**  **Pandemic year** | **Lower** | **Upper** |
| No Previous NCD Morbidity | 11.85 | 8.12 | 17.30 |
| Immune System-Related | 3.47 | 2.92 | 4.13 |
| Cardiovascular | 3.36 | 3.17 | 3.56 |
| Endocrine & Metabolic | 3.26 | 3.05 | 3.49 |
| Cancer | 3.18 | 2.92 | 3.46 |
| 3 or More Morbidities | 2.95 | 2.77 | 3.14 |
| Mental Disorders | 2.62 | 2.46 | 2.80 |
| Chronic Respiratory Diseases | 2.70 | 2.49 | 2.92 |
| Chronic Kidney Diseases | 2.75 | 2.50 | 3.02 |
| Chronic Liver Diseases | 2.58 | 2.14 | 3.11 |
| Substance Use Disorders | 2.17 | 1.90 | 2.48 |

# Table 4. Annual total deaths and population in British Columbia, Canada in the past decade.^4^

| **Year** | **Total Deaths** | **Total Population** |
| --- | --- | --- |
| 2011 | 32,042 | 4,502,104 |
| 2012 | 32,656 | 4,566,769 |
| 2013 | 33,309 | 4,630,077 |
| 2014 | 33,942 | 4,707,103 |
| 2015 | 35,355 | 4,776,388 |
| 2016 | 36,784 | 4,859,250 |
| 2017 | 38,683 | 4,929,384 |
| 2018 | 38,652 | 5,010,476 |
| 2019 | 38,732 | 5,094,796 |
| 2020 | 41,464 | 5,155,495 |
| 2021 | 44,720 | 5,202,378 |

# **References**

1. World Health Organization. International classification of diseases: ninth revision, basic tabulation list with alphabetic index. World Health Organization, 1978.

2. Canadian Institute for Health Information. International statistical classification of diseases and related health problems, tenth revision, Canada (ICD-10-CA). Ottawa, ON: Canadian Institute for Health Information, 2018.

3. BC Ministry of Health Chronic Disease Information Working Group. 2018. BC chronic disease and selected procedure case definitions. https://www.bccdc.ca/Our-Services-Site/Documents/depression.pdf Accessed 8 Sept 2021.

4. British Columbia Ministry of Health [creator]. Vital Events Deaths. V2. 2022. Canada: Population Data BC [publisher]. Data Extract.
